# Enantioselectivity and Enzyme-Substrate Docking Studies of a Ketoreductase from *Sporobolomyces salmonicolor* (SSCR) and *Saccharomyces cerevisiae* (YOL151w)

**DOI:** 10.1155/2014/124289

**Published:** 2014-08-17

**Authors:** Phung-Hoang Nguyen, Maya West, Brent D. Feske, Clifford W. Padgett

**Affiliations:** Armstrong State University, Savannah, GA 31419, USA

## Abstract

Models for two ketoreductases were created and used to predict the stereoselectivity of the enzymes. One was based on the crystal structure of *Sporobolomyces salmonicolor*. This model was used to predict the stereoselectivity for 46 ketone reductions using this enzyme; only 6 were incorrectly predicted. The stereochemistries of the products were compared to the experimental values found in the literature. The Prelog rules were also used to predict the stereoselectivity for this enzyme; however the Prelog rules seem to be highly substrate dependent. As a result, predicting stereoselectivity of KREDs is more complicated than is allowed for with just substrate size and geometry. This enzyme showed Prelog docking geometry for 13 substrates if the enzyme is assumed to prefer an anti-Prelog docking geometry. For SSCR the molecular modeling proved to be a better method for predicting stereoselectivity of the enzymes. The second model was a homology model for YOL151w based on the enzyme crystal structure of *Sporobolomyces salmonicolor* carbonyl reductase, SSCR. In this homology model, 14 compounds were docked and the predicted stereochemistry was compared to the literature values. Of these, 5 were incorrectly predicted.

## 1. Introduction

In 1999, 33% of all dosage-form drug sales in the USA were of single enantiomers [1] and in 2006 that number had risen to 75% [2]. As the use of chiral active pharmaceutical ingredients (APIs) grows, the need for asymmetric synthetic strategies has grown as well. The utilizations of chiral separation and asymmetric starting materials are still popular strategies; however, the fastest growing strategy has been the use of enzymes as an asymmetric catalyst [3]. One of the most popular biocatalyst classes has been ketoreductases (KREDs), which have been popularly used as an asymmetric catalyst to reduce prochiral ketones into optically pure alcohols [4]. These enzymes were initially used by adding the ketone substrate to wild type living cells (such as bakers' yeast (*Saccharomyces cerevisiae*)) and these cells would supply the reduced cofactor (NAD(P)H) along with the KREDs [5]. Since yeast contains many active KREDs, Stewart mined the bakers' yeast genome and developed a library of KREDs overexpressed in* E. coli* [6]. This library has allowed for the direct screening and characterization of each enzyme with several ketoesters and ketonitrile substrates [7, 8] and has led to biocatalytic products with better and opposite stereoselectivities than what has been reported by wild type bakers' yeast. The most promiscuous KRED from this enzyme library has the gene name GRE2 and is often referred to by its yeast open reading frame, YOL151w. Since this enzyme is the most promiscuous KRED in this library and it often affords alcohols with high enantiomeric excess, it can be a very useful asymmetric catalyst for the synthetic chemist. Therefore, a computer model that can accurately model the enzyme-substrate complex of YOL151w would be a very advantageous.

Recently Zhu et al. thoroughly studied a carbonyl reductase from red yeast (*Sporobolomyces salmonicolor*) [9]. This carbonyl reductase (designated as SSCR) was screened and shown to asymmetrically reduce 46 different prochiral ketones. This synthetically useful enzyme was chosen because its structure had been previously determined using X-ray crystallography (PDB ID: 1Y1P) [10] and thus it was a good candidate for molecular docking studies. Their computational model based on the crystal structure of the enzyme was used to accurately predict the enantioselectivity of 11 substrates; no simulation data was reported for the other 35 substrates. In this study, we docked all 46 substrates [9] for which experimental data was available and compared the predicted stereochemistry to that of the experimental values.

There are two main goals of this work. One was to use the X-ray crystal structure of SSCR to build a model that could predict most of the stereoselectivity seen for this enzyme in the literature. As SSCR is a highly promiscuous enzyme that often results in high stereoselectivity, elucidating its behavior can increase its value as a tool for asymmetric synthesis. The second goal is to use a homology model for YOL151w to predict the stereoselectivity of this enzyme for new substrates of interest. In this paper we discuss the modeling work on SSCR and the homology model for YOL151w built from the X-ray structure of SSCR.

## 2. Computational Methods

### 2.1. Docking Simulations of SSCR

The 3D X-ray structure of SSCR was obtained from the Protein Data Bank (ID: 1Y1P). There are two molecules in the asymmetric unit of the crystal structure of SSCR, both nearly identical in geometry; as a result only subunit B (following the procedure laid out by Cundari et al.) [11] along with the cofactor (NADPH) and crystallographic water molecules were used in the modeling. The enzyme was solvated with a 6 Å layer of water; hydrogen atoms were added using the protonate 3D [12] algorithm in MOE [13]. The enzyme was minimized using the AMBER99 force field with Marsili-Gasteiger atomic charge [14] and the reaction field treatment of electrostatic interactions. Crystallographic water molecules in the active site were deleted (ones within 7 Å of the hydride source on NADPH) to allow space for substrate docking.

Substrates were drawn and minimized using the Hartree-Fock method with a 6-31G^*^ basis set as implemented in Spartan 06 program [15]. Substrates were docked in MOE; both substrates and the active site amino acid side chains were treated as flexible with the initial substrate conformation obtained from the gas phase minimum. The active site was chosen to be all residues within 7.5 Å of the hydride source of the cofactor. The substrates were docked in the active site using Triangle Matcher [13] as the placement method; 10000 poses were tried and the London dG scoring function [13] was used to select the best 100 docked poses. Those were further optimized by an untethered force field refinement which allowed the residues in the active site to move. Docked structures were rejected if the carbonyl carbon atom was more than 4 Å from the hydride source or the carbonyl oxygen atom was not within 3.1 Å of two of the three hydrogen-bonding catalytic residues (SER133, TYR177, and LYS181) [9]. The rationale for discarding these structures was that reduction would not occur under those conditions. The docking of each substrate was performed three times and the data sets were combined for analysis; in general only one docking run was necessary as they produced very similar low energy poses.

### 2.2. Homology Model and Docking Simulations of YOL151w

The homology model of YOL151w was built based on the enzyme crystal structure of* Sporobolomyces salmonicolor *carbonyl reductase (Protein Data Bank Code: 1Y1P) that is 31% identical and 17% similar to YOL151w and was the closest match found using the WU-BLAST feature of the* Saccharomyces* Genome Database [16]. The model was built using HHpred/HHsearch and MODELLER software [17–19] then optimized using foldX in Yasaer [20]. The homology model was optimized in MOE via a series of minimizations. Each was run until the root mean squared gradient fell below 0.1 kcal. First, heavy atoms were tethered to 10,000; successive minimizations slowly removed the tethered atoms (1000, 500, and 100) until the tether was removed. The model was validated using Molprobity [21], which gave a clash score for all atoms of 1.49; the MolProbity score was 2.33. The RMSD between the homology model for YOL151w and the X-ray structure was 1.698 Å for all atoms and 1.475 Å for the atoms in the backbone. Sequence alignment generated by HHPred for the homology model is provided in the supplemental materials. The cofactor NADPH was added using MOE, by aligning the 1Y1P structure with the homology model for YOL151w and transferring the cofactor, followed by a series of minimizations with heavy atoms tethered so the cofactor could be inserted into the homology model without drastically altering the geometry of the cofactor or the homology model. The homology model was solvated with a 6 Å layer of water; hydrogen atoms were added using the protonate 3D [12] algorithm in MOE, and the enzyme was minimized using the AMBER99 force field with Marsili-Gasteiger atomic charge and the reaction field treatment of electrostatic interactions. Water molecules in the active site were deleted (ones within 7 Å of the hydride source on NADPH). Substrates were created and docked as described above. Docked structures in which the carbonyl carbon was more than 4 Å from the hydride source or the carbonyl oxygen was not hydrogen-bonded to two amino acids that are part of the active site (HIS212 SER127, TYR165 determined by comparison to SSCR) were discarded since reduction could not occur under those conditions (within 4.1 Å). The docking algorithm used was the same as for SSCR; see above.

## 3. Results and Discussion

### 3.1. SSCR Docking with Substrates

**Scheme 1:**
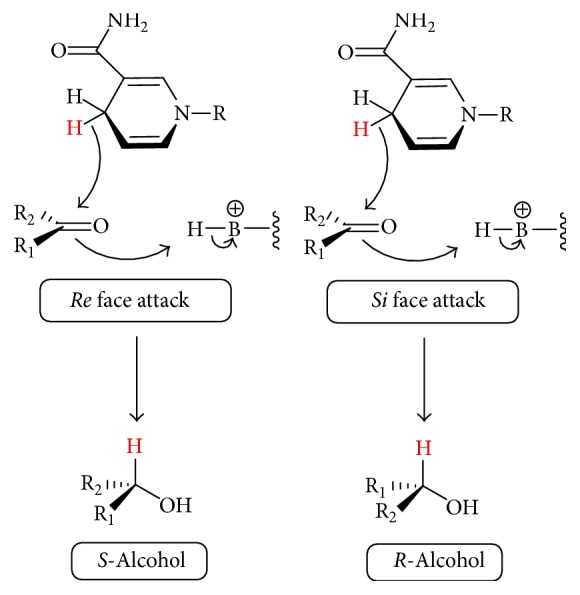
Mechanism of stereoselectivity for NAD(P)H-dependent ketoreductase (1Y1P) and homology model of YOL151w. Group priorities are based on Cahn-Ingold-Prelog rules and assumed in the schemeto be OH > R_1_ > R_2_. B represents any residue capable of donating a hydrogen atom.

In the analysis of the SSCR enzyme, substrates were grouped into four classes based on the functional groups they possess. Examination of the lowest energy structure that met the docking criteria (see above) was performed to determine the stereochemistry that would result from the docking; this was compared to the experimental literature results. Stereochemistry was determined from docking geometry by examining the orientation of the carbonyl group in relation to the hydride source on the cofactor. The docked geometry allowed for determination of the face (Re/Si) that would be attacked by the hydride on the cofactor and thus for prediction of the stereochemistry of the product (see Scheme 1).

**Figure 1:**
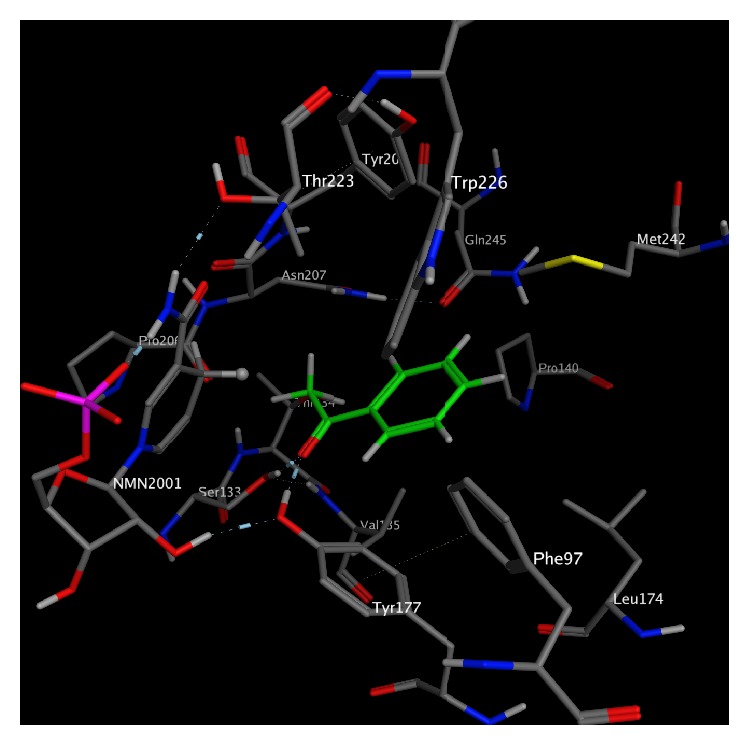
Lowest energy complex of SSCR (1Y1P) and ArK1. ArK1 is colored green, and the hydrogen on NADPH involved with reduction is shown as a ball.

Data from the docking of 26 aryl ketones (ArKs) are shown in Table 1 for the carbonyl reductase (SSCR) [9, 11].  Figure 1 shows the docked enzyme-substrate complex for ArK1. Of these 26 substrates only two had their stereochemistry incorrectly predicted (ArK18 and ArK22) by the model. The model was compared to literature values (obtained via experiment); see the caption of each table for literature references. Similarly, Table 2 shows five aliphatic ketones (ApK) that were simulated and our model correctly predicts three of them.  Table 3 displays *α*-keto esters (AKE) of which there are nine examples [9] from the literature and our model correctly predicts eight of them. Finally, Table 4 shows *β*-ketoesters (BKE) of which six substrates were simulated and compared to their experimental designation and our model correctly predicts 5 of them. Docking energy errors in tables are in the order of 0.5 kcal/mol. In all tables *E*_R_ and *E*_S_ refer to the lowest energy docking pose that meet the criteria for valid structure laid out above; *E*_R_ is the energy of docking pose resulting in a new R chiral center; and *E*_S_ is the same for an S chiral center. The literature values for the enantiomeric excess (ee%) (obtained experimentally) are also shown in the tables as well as the enzyme Prelog behavior for the given substrate (further described below). In the table NS means no structure was found meeting all the criteria listed above for enzyme chemistry to occur.

**Scheme 2:**
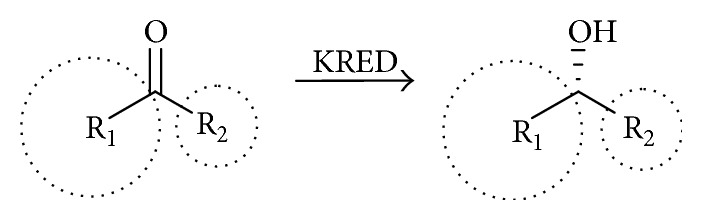
Asymmetric reduction of ketones according to the anti-Prelog rule for discrimination of the faces of carbonylic groups by the enzymes. Note: R_1_ is larger in size than R_2_.

The stereoselectivity of carbonyl reductases can often be predicted by Prelog's rule, which states that the stereochemistry can be determined by looking at the size of the two R groups. This rule states that the enzyme has a large and small pocket that makes up the active site in which the substrate binds and controls the stereochemistry of the product based on the geometry of the substrate. The SSCR enzyme seems to follow the anti-Prelog rule (as was noted by others [22] and in related enzymes [23, 24]). When a ketone substrate binds, its larger R_1_ group is bound in the large pocket and the smaller R_2_ group in the smaller pocket. Then the hydride source of the cofactor attacks from above resulting in an alcohol that has been pushed “back,” Scheme 2, corresponding to anti-Prelog rule behavior.

This enzyme displays the anti-Prelog rule 21 out of 26 times for the ArKs (Table 1), providing an explanation for why the stereochemistry is seen to reverse in the series ArK1 to ArK6. Note the size of the R groups was based off the volume of the R group as calculated in Spartan from the lowest energy gas phase conformation of the substrates [15]. In this series, one R-group is a phenyl while the other is an n-alkyl group (where n is the same as the ArK number). Between ArK2 and ArK3 the stereochemistry reverses, as shown by experiment. The docking model also predicts a reversal in the stereochemistry as the n-alkyl group length increases. Compounds ArK18, ArK25, and ArK26 show a deviation from the anti-Prelog behavior of this enzyme.

In the docking simulations of the ArKs with SSCR, the carbonyl oxygen on the ArKs participates in hydrogen bonding with SER133 and TYR177 for all of the complexes. The energies generally show that the lowest energy geometry leads to the observed stereochemistry. The docking model predicts the wrong stereochemistry for ArK18 and ArK22. Both ArK18 and ArK22 have halogen atoms present and the docking model may be poorly reproducing the interaction that is occurring with the halogen atoms. The correlation between enantiomeric excess and energy difference seen in the model is 0.62, and there is an even better correlation between lowest energy conformation and major enantiomer seen experimentally; the correlation value is 0.74. Using the lowest energy docked geometry, more of the stereochemistry of the products was correctly predicted than that obtained from simply using the Prelog rule (noting that this enzyme displays anti-Prelog behavior).

While SSCR has anti-Prelog rule behavior with ArKs, it is interesting to note that it has Prelog rule stereoselectivity with the ApK substrates (Table 2). Only ApK3 shows anti-Prelog behavior; however, the enzyme shows nearly no stereochemical selectivity towards this compound. In this class of compounds, the docking results predicted the stereochemistry incorrectly for two of the five compounds (ApK1 and ApK2) and perhaps this could be attributed to the low stereoselectivity observed from the enzyme.

In the results for the AKEs (Table 3), only one (AKE3) out of the nine substrates did the docked structure predict incorrect stereoselectivity. For these substrates SSCR predominately followed the anti-Prelog rule, whereas only two (AKE8 and AKE9) demonstrated Prelog behavior. However, the size of the two R groups in AKE8 and AKE9 were nearly identical (88 Å^3^ and 82 Å^3^ for AKE8) making the application of Prelog's rule difficult.

The BKEs showed primarily anti-Prelog behavior with two exceptions: BKE4 and BKE6. Again, with these molecules the sizes of the two R-groups on each side of the carbonyl are approximately the same. For the model predictions, only the lowest-energy docked structure for BKE2 predicted the incorrect stereoselectivity. The energies between the pro-R and pro-S docked structure were very close, but there is no clear reason why the docked simulation did not prefer one over the other.

Overall 46 compounds were docked and the predicted stereochemistry was compared to the literature values. Using the lowest energy geometries that are also capable of undergoing reaction (ones whose geometry had the carbonyl group close enough to the hydride source and was close enough to hydrogen bond to two of the catalytic residues), only 6 were incorrectly predicted compared to 13 if the enzyme is assumed to prefer an anti-Prelog docking geometry. Half of the incorrectly predicted stereochemistries were on compounds containing halogen atoms, which may indicate a weakness in the model for highly electronegative atoms.

### 3.2. YOL151w Homology Model Docking Results

As with the modeling of SSCR, substrates were grouped into classes based on their functionality and docked in the homology model of YOL151w. Examination of the lowest-energy structure in which the docking criteria were met was performed to determine what stereochemistry would result from the docking. This predicted stereoselectivity was then compared to the experimental literature results.

The first group of substrates contained seven *β*-ketonitriles (BKN) (Table 5) that were docked in YOL151w [25]. Of these seven compounds, five were accurately modeled and two, BKN6 and BKN7, had their stereochemistry incorrectly predicted by the homology model. For BKN7 there were no structures found to meet the geometry criteria necessary for a reaction to occur (within 4 Å from the hydride source and within 4.1 Å of the two hydrogen-bond donors on amino acids that are part of the catalytic triad). With all of these compounds, YOL151w demonstrated Prelog behavior.

Data from the docking of AKE are shown in Table 6 for YOL151w. Three examples found in the literature were simulated [26]. Of these 3 compounds only AKE10 had its stereochemistry incorrectly predicted by the homology model. There is no clear reason why the stereochemistry of AKE10 was incorrectly predicted. YOL151w demonstrated Prelog behavior only for AKE10 and for the other two it demonstrated anti-Prelog behavior. The next class of substrates investigated was BKE (Table 7). Of the four examples that were simulated [26], two compounds (BKE2 and BKE8) had their stereochemistry incorrectly predicted by the homology model. All of these substrates followed Prelog's rule.

Overall for the homology model, 14 compounds were docked and the predicted stereochemistry was compared to the literature values. Using the lowest energy geometries that are also capable of undergoing reaction, 5 were incorrectly predicted. The correlation between enantiomeric excess and energy difference seen in the model is 0.01, and there is a correlation between the lowest energy conformation and major enantiomer seen experimentally; the correlation values is 0.45. This is about twice the failure rate of the model based on the crystal structure of SSCR and is an indication that the homology model is not as reliable as using a known structure. As a result, the determination of the crystal structure of YOL151w would be a significant advancement for modeling this highly promiscuous and synthetically useful enzyme.

## 4. Conclusion

Two KRED computational models were developed and used to predict the enzyme (SSCR and YOL151w) stereoselectivity for a variety of substrates. For SSCR the crystal structure (PDB ID: 1Y1P) was used to develop the model used in the docking studies. This model proved adequate for predicting the stereochemistry of docked substrates, especially for nonhalogen containing substrates. While predicting the major enantiomer was generally successful, the model could not predict the enantiomeric excess. The second model was a homology model for YOL151w that was based on the crystal structure of the related enzyme (SSCR, 1Y1P). This model was less successful at predicting the stereochemistry resulting from the reduction of carbonyl groups in the enzyme. This is not surprising as building the homology model adds another opportunity for deviations between reality and the model to occur. We plan to build the model for YOL151w from the X-ray structure when it becomes available (attempts are currently being made to obtain the structure).

## Supplementary Material

Sequence alignment generated by HHPred for the homology model, for more information visit

## Figures and Tables

**Table 1:** Aryl ketones (ArK).

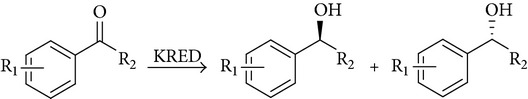								
ID name	Compound name	R_1_	R_2_	*E*_R_ (kcal/mol)	*E*_S_ (kcal/mol)	ee (%)	Prelog	Prediction correct

ArK1∗	Acetophenone	H	CH_3_	−39.9	−37.8	42 (R)	Anti	Y
ArK2∗	Propiophenone	H	CH_2_CH_3_	−40.6	−38.1	28 (R)	Anti	Y
ArK3∗	1-Phenylbutan-1-one	H	(CH_2_)_2_CH_3_	−44.2	−44.4	88 (S)	Prelog	Y
ArK4∗	1-Phenylpentan-1-one	H	(CH_2_)_3_CH_3_	−45.5	−47.3	87 (S)	Prelog	Y
ArK5∗	1-Phenylhexan-1-one	H	(CH_2_)_4_CH_3_	−47.0	−50.7	34 (S)	Anti	Y
ArK6∗	1-Phenylheptan-1-one	H	(CH_2_)_5_CH_3_	−49.7	−53.1	27 (S)	Anti	Y
ArK7∗	2-Methyl-1-phenylpropan-1-one	H	CH(CH_3_)_2_	−44.4	−41.5	98 (R)	Anti	Y
ArK8∗	2,2-Dimethyl-1-phenylpropan-1-one	H	C(CH_3_)_3_	−47.6	NS	98 (R)	Anti	Y
ArK9^†^	2-Chloro-1-phenylethanone	H	CH_2_Cl	−39.9	−45.3	98 (S)	Anti	Y
ArK10∗	Cyclopropyl(phenyl)methanone	H	*cyclo*-C_3_H_5_	−46.2	−41.5	96 (R)	Anti	Y
ArK11∗	Cyclopropyl(4-fluorophenyl)methanone	4′-F	*cyclo*-C_3_H_5_	−49.8	NS	98 (R)	Anti	Y
ArK12∗	4-Chlorophenyl(cyclopropyl)methanone	4′-Cl	*cyclo*-C_3_H_5_	−52.2	−33.7	98 (R)	Anti	Y
ArK13^†^	1-(4-Fluorophenyl)ethanone	4′-F	CH_3_	−40.4	−36.2	46 (R)	Anti	Y
ArK14^†^	1-(4-Chlorophenyl)ethanone	4′-Cl	CH_3_	−44.7	NS	14 (R)	Anti	Y
ArK15^†^	1-(4-Bromophenyl)ethanone	4′-Br	CH_3_	−44.6	NS	42 (R)	Anti	Y
ArK16^†^	1-*p*-Tolylethanone	4′-CH_3_	CH_3_	−42.7	NS	59 (R)	Anti	Y
ArK17^†^	1-(4-Methoxyphenyl)ethanone	4′-OCH_3_	CH_3_	−42.9	NS	57 (R)	Anti	Y
ArK18^†^	1-(4-(Trifluoromethyl)phenyl)ethanone	4′-CF_3_	CH_3_	−46.7	NS	17 (S)	Prelog	N
ArK19^†^	1-(2-chlorophenyl)ethanone	2′-Cl	CH_3_	−42.6	−37.6	15 (R)	Anti	Y
ArK20^†^	1-*o*-Tolylethanone	2′-CH_3_	CH_3_	−43.3	−41.3	70 (R)	Anti	Y
ArK21^†^	1-(2-Methoxyphenyl)ethanone	2′-OCH_3_	CH_3_	−49.6	−49.5	99 (R)	Anti	Y
ArK22^†^	1-(3-Chlorophenyl)ethanone	3′-Cl	CH_3_	−40.5	−40.8	66 (R)	Anti	N
ArK23^†^	1-*m*-Tolylethanone	3′-CH_3_	CH_3_	−42.3	NS	92 (R)	Anti	Y
ArK24^†^	1-(3,5-Bis(trifluoromethyl)phenyl)ethanone	3′,5′-(CF_3_)_2_	CH_3_	−45.2	NS	99 (R)	Anti	Y
ArK25^†^	1-Tetralone			−42.4	−37.7	94 (R)	Prelog	Y
ArK26^†^	6-Methyl-4-chromanone			−46.2	−45.7	99 (R)	Prelog	Y

NS = no structure found meeting the requirements. *E*_R_ and *E*_S_ refer to the lowest energy docking pose that meets the criteria for valid structure whose geometry is pro R or S, respectively. Literature values for the enantiomeric excess (ee (%)) were obtained as follows: ∗values are from [11], and ^†^values are from [9]. Prelog column indicates if the enzyme followed prelog or antiprelog rule for the given substrate. The last column indicates if the model correctly predicted the experimental results.

**Table 2:** Aliphatic ketones (ApK).

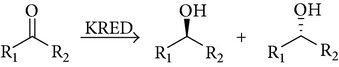								

ID name	Compound name	R_1_	R_2_	*E*_R_ (kcal/mol)	*E*_S_ (kcal/mol)	ee (%)	Prelog	Prediction correct

ApK1	Heptan-2-one	n-Pentyl	CH_3_	−43.3	−41.2	30 (S)	Prelog	N
ApK2	Octan-2-one	n-Hexyl	CH_3_	−46.8	NS	44 (S)	Prelog	N
ApK3	Nonan-2-one	n-Heptyl	CH_3_	−47.3	NS	4 (R)	Anti	Y
ApK4	1-Adamatyl methyl ketone	1-Adamantyl	CH_3_	−45.1	−46.9	>99 (S)	Prelog	Y
ApK5	Octane-3-one	n-Pentyl	CH_2_CH_3_	−45.1	−42.5	72 (R)	Prelog	Y

NS = no structure found meeting the requirements. *E*_R_ and *E*_S_ refer to the lowest energy docking pose that meets the criteria for valid structure whose geometry is pro R or S, respectively. Literature values for the enantiomeric excess (ee (%)) were obtained from [9]. Prelog column indicates if the enzyme followed prelog or antiprelog rule for the given substrate. The last column indicates if the model correctly predicted the experimental results.

**Table 3:** Alpha-ketoesters (AKE).

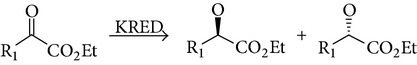							

ID name	Compound name	R_1_	*E*_R_ (kcal/mol)	*E*_S_ (kcal/mol)	ee (%)	Prelog	Prediction correct

AKE1	Ethyl 2-oxo-2-phenylacetate	Phenyl	−51.4	−51.7	99 (S)	Anti	Y
AKE2	Ethyl 2-(4-cyanophenyl)-2-oxoacetate	4-Cyanophenyl	−54.4	−64.7	82 (S)	Anti	Y
AKE3	Ethyl 2-(4-fluorophenyl)-2-oxoacetate	4-Fluorophenyl	−53.8	−39.6	74 (S)	Anti	N
AKE4	Ethyl 2-(4-chlorophenyl)-2-oxoacetate	4-Chlorophenyl	NS	−47.9	63 (S)	Anti	Y
AKE5	Ethyl 2-(4-bromophenyl)-2-oxoacetate	4-Bromophenyl	−52.2	−55.9	56 (S)	Anti	Y
AKE6	Ethyl 2-oxo-2-*p*-tolylacetate	4-Methylphenyl	−53.1	−55.0	88 (S)	Anti	Y
AKE7	Ethyl 2-(3,5-difluorophenyl)-2-oxoacetate	3,5-Difluorophenyl	NS	−41.7	43 (S)	Anti	Y
AKE8	Ethyl 4-methyl-2-oxopentanoate	Isopropyl	−50.2	−44.7	99 (R)	Prelog	Y
AKE9	Ethyl 4,4-dimethyl-2-oxopentanoate	*tert*-Butyl	−54.5	−46.4	99 (R)	Prelog	Y

NS = no structure found meeting the requirements. *E*_R_ and *E*_S_ refer to the lowest energy docking pose that meets the criteria for valid structure whose geometry is pro R or S, respectively. Literature values for the enantiomeric excess (ee (%)) were obtained from [9]. Prelog column indicates if the enzyme followed prelog or antiprelog rule for the given substrate. The last column indicates if the model correctly predicted the experimental results.

**Table 4:** Beta-ketoesters (BKE).

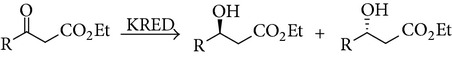							

ID name	Compound name	R	*E*_R_ (kcal/mol)	*E*_S_ (kcal/mol)	ee (%)	Prelog	Prediction correct

BKE1	Ethyl 4-chloro-3-oxobutanoate	Chloromethyl	−46.8	−61.2	97 (S)	Anti	Y
BKE2	Ethyl 3-oxopentanoate	Ethyl	−50.2	−50.3	61 (R)	Anti	N
BKE3	Ethyl 4-methyl-3-oxopentanoate	Isopropyl	−52.0	−63.9	99 (S)	Anti	Y
BKE4	Ethyl 4,4-dimethyl-3-oxopentanoate	*tert*-Butyl	−47.6	−59.5	99 (S)	Prelog	Y
BKE5	Ethyl 4,4,4-trifluoro-3-oxopentanoate	Trifluoromethyl	−43.1	−58.4	90 (S)	Anti	Y
BKE6	Ethyl 3-oxo-3-phenylpropanoate	Phenyl	−54.4	−57.8	56 (S)	Prelog	Y

NS = no structure found meeting the requirements. *E*_R_ and *E*_S_ refer to the lowest energy docking pose that meets the criteria for valid structure whose geometry is pro R or S, respectively. Literature values for the enantiomeric excess (ee (%)) were obtained from [9]. Prelog column indicates if the enzyme followed prelog or antiprelog rule for the given substrate. The last column indicates if the model correctly predicted the experimental results.

**Table 5:** Beta-ketonitrile (BKN).

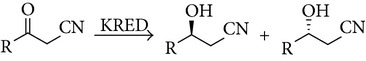							

ID name	Compound name	R	*E*_R_ (kcal/mol)	*E*_S_ (kcal/mol)	ee (%)	Prelog	Prediction correct

BKN1	5-Methyl-3-oxohexanenitrile	Isobutyl	−46.2	NS	99 (R)	Prelog	Y
BKN2	3-Cyclohexyl-3-oxopropanenitrile	Hexyl	NS	−38.2	99 (S)	Prelog	Y
BKN3	3-Oxo-3-phenylpropanenitrile	Phenyl	NS	−48.1	99 (S)	Prelog	Y
BKN4	3-(4-Fluorophenyl)-3-oxopropanenitrile	4-fluorophenyl	NS	−62.5	99 (S)	Prelog	Y
BKN5	3-(4-Chlorophenyl)-3-oxopropanenitrile	4-chlorophenyl	NS	−41.3	78 (S)	Prelog	Y
BKN6	3-(4-Methoxyphenyl)-3-oxopropanenitrile	4-methoxyphenyl	−43.6	NS	99 (S)	Prelog	N
BKN7	Methyl 4-(2-cyanoacetyl)benzoate	2-cyanoacetyl	NS	NS	74 (S)	Prelog	N

NS = no structure found meeting the requirements. *E*_R_ and *E*_S_ refer to the lowest energy docking pose that meets the criteria for valid structure whose geometry is pro R or S, respectively. Literature values for the enantiomeric excess (ee (%)) were obtained from [7]. Prelog column indicates if the enzyme followed prelog or antiprelog rule for the given substrate. The last column indicates if the model correctly predicted the experimental results.

**Table 6:** Alpha-ketoesters (AKE).

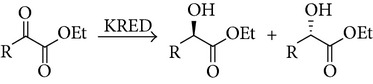							

ID name	Compound name	R	*E*_R_ (kcal/mol)	*E*_S_ (kcal/mol)	ee (%)	Prelog	Prediction correct

AKE10	Ethyl 2-oxobutanoate	Ethyl	−54.4	−42.3	52 (S)	Prelog	N
AKE11	Ethyl 2-oxopentanoate	n-Propyl	−46.5	−46.2	98 (R)	Anti	Y
AKE12	Ethyl 2-oxo-4-phenylbutanoate	PhCH_2_CH_2_	NS	−56.3	98 (S)	Anti	Y

NS = no structure found meeting the requirements. *E*_R_ and *E*_S_ refer to the lowest energy docking pose that meets the criteria for valid structure whose geometry is pro R or S, respectively. Literature values for the enantiomeric excess (ee (%)) were obtained from [26]. Prelog column indicates if the enzyme followed prelog or antiprelog rule for the given substrate. The last column indicates if the model correctly predicted the experimental results.

**Table 7:** Beta-ketoesters (BKE).

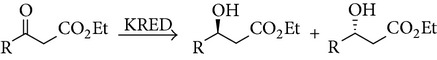							

ID name	Compound name	R	*E*_R_ (kcal/mol)	*E*_S_ (kcal/mol)	ee (%)	Prelog	Prediction correct

BKE1	Ethyl 4-chloro-3-oxobutanoate	Chloromethyl	−46.3	−34.8	98 (R)	Prelog	Y
BKE2	Ethyl 3-oxopentanoate	Ethyl	−55.3	−47.3	98 (S)	Prelog	N
BKE7	Ethyl 3-oxobutanoate	Methyl	−51.2	−52.0	98 (S)	Prelog	Y
BKE8	Ethyl 3-oxohexanoate	n-Propyl	−56.5	−47.9	98 (S)	Prelog	N

NS = no structure found meeting the requirements. *E*_R_ and *E*_S_ refer to the lowest energy docking pose that meets the criteria for valid structure whose geometry is pro R or S, respectively. Literature values for the enantiomeric excess (ee (%)) were obtained from [26]. Prelog column indicates if the enzyme followed prelog or antiprelog rule for the given substrate. The last column indicates if the model correctly predicted the experimental results.
