# Supplementary material for: Enantioselectivity and Enzyme-Substrate Docking Studies of a Ketoreductase from Sporobolomyces salmonicolor (SSCR) and Saccharomyces cerevisiae (YOL151w)
Source: Int Sch Res Notices. 2014 Aug 17;2014:124289. doi: 10.1155/2014/124289 (PMC4897451; doi:10.1155/2014/124289)
Supplement: Supplementary file 1 — Sequence alignment generated by HHPred for the homology model, for more information visit [file 124289.f1.pdf]

```
1y1p_A ARII, aldehyde reductase II; rossmann fold, short chain dehydrogenase reductase, oxidoreductase;

HET: NMN AMP; 1.60A {Sporidiobolus salmonicolor} SCOP: c.2.1.2 PDB: 1ujm_A* 1zze_A

Probab=100.00  E-value=3.6e-40  Score=298.98  Aligned_cols=318  Identities=30%  Similarity=0.433  Sum_probs=0.0

Q ss_pred             CeEEEECCchHHHHHHHHHHHHHCCCEEEEEeCCHHHHHHHHHHhcccC--CceEEEEeccCCCchhHHHHHhhccCCCcEE
Q no_name_0         1 MSVFVSGANGFIAQHIVDLLLKEDYKVIGSARSQEKAENLTEAFGNN--PKFSMEVVPDISKLDAFDHVFQKHGKDIKIV   78 (342)
Q Consensus         1 ~~vlItGatG~iG~~l~~~L~~~g~~V~~~~R~~~~~~~~~~~~~~~~--~~~~~~~~~~Dl~~~~~~~~~~~~~~~~~~d~v   78 (342)
                      |+|||||||||++|+++|+++||+|+++.|+.++...+.......  ......+.+|+.|.+.+.+++    ..+|.|
T Consensus        12 k~VlVTGatGfIGs~lv~~Ll~~G~~V~~lvR~~~~~~~~~~~~~~~~~~~~~~~~~~~~Di~d~~~~~~~~~~----~~~~~v   87 (342)
T 1y1p_A         12 SLVLVTGANGFVASHVVEQLLEHGYKVRGTARSASKLANLQKRWDAKYPGRFETAVVEDMLKQGAYDEVI----KGAAGV   87 (342)
T ss_dssp             CEEEEETTTSHHHHHHHHHHHHHTTCEEEEEESSHHHHHHHHHHHHHHSTTTEEEEECSCTTSTTTTTTTT----TTCSEE
T ss_pred             CEEEEECCCcHHHHHHHHHHHHHCcCEEEEEeCChhHHHHHHHhhcccccccccEEEecccCHHHHhhh----ceeeEE


Q ss_pred             EEcCCCcCCCCCChHHHHHHHHHHHHHHHHHHHHHHhCCCCcCEEEEeCcHHHhcCCccccCCCCceeCcccCCCcchhhh-
Q no_name_0        79 LHTASPFCFDITDSERDLLIPAVNGVKGILHSIKKYAADSVERVVLTSSYAAVFDMAKENDKSLTFNEESWNPATWESC-  157 (342)
Q Consensus        79 ih~a~~~~~~~~~~~~~~~~~~~~~~~~nv~~~~~ll~~~~~~~~~~~~~~~~~~v~~SS~~~~~~~~~~~~~~~~~~~~~~e~~~~~~~~~~~~~-  157 (342)
                      +|+|+......  .....+++|+.++.+++++|.+.+ . +++|||+||++ .+............+|+.+.....+..
T Consensus        88 ~~~a~~~~~~~~~--~~~~~~~~~~~nv~~t~n~l~~~~~~~~~-~-i~~~v~~SS~~-~~~~~~~~~~~~~~~~~~e~~~~~~~~~~~~~~~   162 (342)
T 1y1p_A         88 AHIASVVSFSN--KYDEVVTPAIGGTLNALRAAAATP-S-VKRFVLTSSTV-SALIPKPNVEGIYLDEKSWNLESIDKAK  162 (342)
T ss_dssp             EECCCCCSCCS--CHHHHHHHHHHHHHHHHHHHHTCT-T-CCEEEEECCGG-GTCCCCTTCCCCEECTTCCCHHHHHHHH
T ss_pred             eeeccccccc--chhHHHhHHHHHHHHHHHHHHhcc-c-ccccccccceE-EEeccCCCccccccccccCccccccc


Q ss_pred             ------ccccchhHHHHHHHHHHHHHHHHHHhCCCCCCeEEEEEeCCcccCCCCccccccccccccccccccHHHHHHHHHHhCCCCCC
Q no_name_0       158 ------QSDPVNAYCGSKKFAEKAAWEFLEENRDSVKFELTAVNPVYVFGPQMFDKDVKKHLNTSCELVNSLMHLSPEDK  231 (342)
Q Consensus       158 ------~~~p~~~Y~~sK~~~E~~~~~~~~~~~~~~~~~~~~~~g~~~~~~lrp~~v~g~~~~~~~~~~~~~~~~~~~~~~~~~~~~~~~~~~~~~~~~~~~  231 (342)
                            +..|.++|+.||.++|.+++.+.+.++.  ++.++++||+.+|||...+.   .....+..++..+++|.....
T Consensus       163 ~~~~~~~~~~~~~~~~~Y~~sK~~~E~~~~~~~~~~~~~~~~~~~~~--~~~~~~~~i~p~~v~Gp~~~~~~---~~~~~~~~~~~i~~~~~g~~~~~   237 (342)
T 1y1p_A        163 TLPESDPQKSLWVYAASKTEAELAAWKFMDENKP--HFTLNAVLPNYTIGTIFDPE---TQSGSTSGWMMSLFNGEVSPA  237 (342)
T ss_dssp             HSCTTSTTHHHHHHHHHHHHHHHHHHHHHHHHHHCC--SSEEEEEEESEEECCCSCTT---TCCCHHHHHHHHHHTTCCCHH
T ss_pred             ccccCCccCCcchHHHHHHHHHHHHHHHHHHHHhCC--CcceEEecCcceECCCcCcc---cccccHHHHHHHHHcCCCCCcc


Q ss_pred             C-cccCCCeeEHHHHHHHHHHHHHcCCCCCCCceEEEeCCC-cCHHHHHHHHHHHCccccccCCCCCCCCCccccccCCcceeec
Q no_name_0       232 I-PELFGGYIDVRDVAKAHLVAFQKRETIGQRLIVSEAR-FTMQDVLDILNEDFPVLKGNIPVGKPGSGATHNTLGATLD  309 (342)
Q Consensus       232 ~-~~~~~~~~~i~v~D~a~a~~~~~~~~~~~~~~~~~g~~~nv~~~~-~s~~e~~~~i~~~~~~~~~~~~~~~i~~~~~~~~~~~~~~~~~~~~~~~~~~~d  309 (342)
                      . +...++|+|++|+|++++.+++++. .++.||++++. +++.|+++.+.+.+|..  .++...+............+
T Consensus       238 ~~~~~~~~~~~~~i~v~D~a~~~i~al~~~~~-~~g~~~~~~~~~~~~~~~t~~ei~~~i~~~~~p~~--~i~~~~~~~~~~~~~~~~~~~~~~~~~~~   314 (342)
T 1y1p_A        238 LALMPPQYYVSAVDIGLLHLGCLVLPQ-IERRRVYGTAGTFDWNTVLATFRKLYPSK--TFPADFPDQGQDLSKFDTAPS  314 (342)
T ss_dssp             HHTCCSEEEEEHHHHHHHHHHHHHHCTT-CCSCEEEECCEEECHHHHHHHHHHHCTTS--CCCCCCCCCCCCCCCCEECCHHH
T ss_pred             cCCCCCceeeeeHHHHHHHHHHHHHhCCC-cCCeEEEEcCCCcEeHHHHHHHHHHHcCCC--cCCCCCCCCCCCCCCcccChhHH


Q ss_pred             cHHHHHHhCC-CCCCHHHHHHHHHHHHHHH
Q no_name_0       310 NKKSKKLLGF-KFRNLKETIDDTASQIL  336 (342)
Q Consensus       310 ~~~~~~~~~~lG~-~~~~~~~~~~~~~~i~~~~~~~~~~~~~~  336 (342)
                      .++++. +|| +.++++|+|+++++|.+
T Consensus       315 ~~~lk~-lg~~~~~~~~~lee~i~d~ies~~  341 (342)
T 1y1p_A        315 LEILKS-LGRPGWRSIEESIKDLVGSET  341 (342)
T ss_dssp             HHHHHH-TTCCSCCCHHHHHHHHHCCSC
T ss_pred             HHHHHH-cCCCCCcCHHHHHHHHHHHCc
```
